# RELIGIOSITY, RELIGIOUS BELIEFS, AND COGNITIVE STATUS AMONG BLACK AND WHITE MEN WITH SIMILAR INCOMES

**DOI:** 10.1093/geroni/igad104.1234

**Published:** 2023-12-21

**Authors:** Marino Bruce, Bettina Beech, Gillian Marshall, Nicole Phillips, Harlan Jones, Corinne Pettigrew, Roland J Thorpe

**Affiliations:** University of Houston/Tilman J. Fertitta Family College of Medicine, Houston, Texas, United States; University of Houston, Houston, Texas, United States; NORC at the University of Chicago, Chicago, Illinois, United States; University of North Texas Health Science Center, Fort Worth, Texas, United States; University of North Texas Health Science Center, Fort Worth, Texas, United States; Johns Hopkins University, Baltimore, Maryland, United States; Johns Hopkins University, Baltimore, Maryland, United States

## Abstract

Black men experience multiple stressors linked with cognitive impairment and have higher risks for Alzheimer’s Disease and related dementias than their White peers. Yet, few studies have focused on coping resources. Religious beliefs and institutions are thought to have health benefits; however, no study to our knowledge has examined religious practices or beliefs and their association with cognitive impairment disparities among Black and White men. The purpose of this study was to examine the association between religious practices and/or beliefs and cognitive status among Black and White men in the Health and Retirement Study (HRS). Data were drawn from Black and White men who reported incomes at or below $50,000 and completed the Core and Leave Behind Questionnaires in the 2016 HRS. The primary outcome was any cognitive impairment, a dichotomous variable derived from a modified version of the Telephone Interview for Cognitive Status (TICS). Religious service attendance, private prayer frequency, and religious beliefs were the primary independent variables. The proportion of Black men was 24.2% and had a larger proportion of individuals with any cognitive impairment than their White peers (47.2% vs 29.4%). Results from race-stratified Modified Poisson Regression models indicated that occasional religious service attendance was beneficial for White men (PR=0.68, CI:0.52-0.89) while private prayer (PR=0.71, CI:0.52-0.98) and religious beliefs (PR=0.90, CI:0.83-0.97) were inversely related to any cognitive impairment among Black men. Full-scale studies of Black men with robust religiosity measures are needed to further examine the association between religious factors and cognitive functioning among this group.

